# Comparing effects of natural betaine and betaine hydrochloride on gut physiology in broiler chickens

**DOI:** 10.1016/j.psj.2022.102173

**Published:** 2022-09-09

**Authors:** Wageha A. Awad, Daniel Ruhnau, Ana Gavrău, Károly Dublecz, Michael Hess

**Affiliations:** ⁎ Clinic for Poultry and Fish Medicine, Department for Farm Animals and Veterinary Public Health, University of Veterinary Medicine, Vienna, Austria; † Agrana Sales & Marketing GmbH, Vienna, Austria; ‡ Institute of Physiology and Nutrition, Georgikon Campus, Hungarian University of Agriculture and Life Science, Keszthely, Hungary

**Keywords:** betaine, natural, synthetic, intestinal permeability, broiler chickens

## Abstract

Betaine is a well-known component of poultry diets with various effects on nutritional physiology. For example, increased water retention due to the osmolytic effect of betaine increases the volume of the cell, thereby accelerating the anabolic activity, integrity of cell membrane, and overall performance of the bird. Betaine is a multifunctional component (trimethyl derivative) acting as the most efficient methyl group donor and as an organic osmolyte, which can directly influence the gastrointestinal tract integrity, functionality, and health. So far, nothing is known about the effect of betaine on the intestinal barrier in chickens. In addition, little is known about comparing natural betaine with its synthetic form. Therefore, an animal study was conducted to ascertain the effects of betaine supplementation (natural and synthetic) on performance and intestinal physiological responses of broilers. One hundred and five 1-day-old broiler chicks were randomly assigned into 3 groups with 35 birds each: control, natural betaine (1 kg active natural (n)-betaine/ton of feed) and synthetic (syn)-betaine‐HCL (1 kg active betaine /ton of feed). Histological assessment showed lower jejunal crypt depth and villi height/crypt depth ratio in syn-betaine-HCL group compared with natural n-betaine fed birds. Furthermore, it was found that syn-betaine-HCL negatively affects the integrity of the intestine by increasing the intestinal paracellular permeability in both jejunum and cecum as evidenced by a higher mannitol flux. Additionally, syn-betaine-HCl significantly upregulated the IFN-γ mRNA expression at certain time points, which could promote intestinal permeability, as it plays an important role in intestinal barrier dysfunction. Body weight (**BW**) and body weight gain (**BWG**) did not differ (*P* > 0.05) between the control birds and birds supplemented with syn-betaine‐HCL. However, the BW and BWG were significantly (*P* < 0.05) improved by the dietary inclusion of n-betaine compared with other treatments. Altogether, the dietary inclusion of n-betaine had a positive effect on performance and did not negatively affect gut paracellular permeability. Furthermore, our results show that syn-betaine-HCl induces changes in the intestine, indicating an alteration of the intestinal histology and permeability. Thus, natural or synthetic betaine has different effects, which needs to be considered when using them as a feed supplement.

## INTRODUCTION

Betaine is a known multifunctional nutrient in poultry nutrition, as an efficient methyl group donor it can replace other methyl group donors such as choline and methionine, which assists birds under heat stress conditions and improves slaughter characteristics (Rao et al., 2011; Shakeri et al., 2018). The major betaine source so far has been betaine anhydrate extracted from sugar beet. Betaine acts as a strong osmolyte, which is important regarding cellular dehydration via minimizing water loss in the intestine (Kettunen et al., 2001). Furthermore, betaine, being involved in the energy metabolism, can reduce the demand for energy-consuming ion pump activity and supports cells to maintain their function during stress periods (Fu et al., 2022).

In poultry production, data on betaine indicate a positive impact on performance, improved carcass composition, reduced litter moisture as well as being beneficial to overcome coccidiosis and stress (Kettunen et al., 2001; Fetterer et al., 2003). The effects of betaine on the host are resolved by various mechanisms such as altered protein synthesis by enhancing the availability of methionine and influencing several physiological functions like membrane synthesis and acetylcholine formation by increasing the availability of choline (Eklund et al., 2005). The same authors reported also that betaine upregulates the synthesis of methylated compounds such as carnitine, which is required for fatty acids transport across the inner mitochondrial membrane where their oxidation occurs.

Betaine affects intestinal cells by increasing their water-binding capacity, stabilizes the structure of the mucosa and reduces coccidial lesions, and increases cell action as indicated by a higher activity of proteolytic enzymes (Xu and Yu, 2000; Kettunen et al., 2001; Eklund et al., 2005; Amerah and Ravindran, 2015). Furthermore, some studies reported that betaine enhances the digestion and absorption of nutrients, stimulates bacterial fermentation of fiber, improves feed conversion ratios, breast meat yield and meat quality, and reduces carcass fat (Eklund et al., 2005; Metzler-Zebeli et al., 2009; Ratriyanto et al., 2010). Betaine may also reduce digestive disorders and mortality under unfavourable conditions such as infections and heat stress, and thereby improve production efficiency. Several studies showed that dietary betaine supplementation could improve the bird's performance and reduce the negative impact of heat stress on cell viability and immune response by improving cell osmoregulation (Wang et al., 2004; Attia et al., 2005; dos Santosa et al., 2019).

Today, betaine can be supplemented as natural betaine, isolated from natural sources; synthetic anhydrous betaine or synthetic betaine-HCl. Scientific data on the comparison between synthetic betaine-HCl and natural betaine are limited, prompting us to examine whether the 2 forms of betaine have different effects on the intestine. Numerous studies showed that betaine had the ability to improve chicken's performance as mentioned above; however, how betaine exert this positive effect is not known. Additionally, the mechanism behind this positive effect of betaine on gut function is lacking.

Applying the Ussing chambers technique allows the quantitative analyses of flux rates or the uptake of defined molecules (e.g., electrolytes or sugars) across the intestinal epithelium, as indicators for intestinal permeability at given sites (Awad et al., 2018). Despite the numerous reports on the use of betaine in poultry production, nothing is known so far about the effect of betaine on the gut epithelial barrier or gut microbiota in chickens. Therefore, this study was conducted to identify possible alterations in the morphological integrity of the gut in response to betaine (natural and betaine-HCL), intestinal permeability, tight junction, mucin, and cytokines mRNA expression, with particular attention paid to performance characteristics.

## MATERIALS AND METHODS

### Ethics Statement

The animal trial was approved by the institutional ethics committee of the University of Veterinary Medicine and licensed by the national authority according to §26 of the Law for Animal Experiments, Tierversuchsgesetz 2012—BGBl. I Nr. 114/2012 (license number GZ 0.461.437V/3b/2020).

### Experimental Design

One hundred and five 1-day-old broiler chicks (males and females) were obtained from a commercial hatchery (Ross-308, Gefluegelhof Schulz, Graz, Austria) and randomly divided into 3 groups (35 birds/5 replicates/group). The birds were housed on wood shavings and had free access to water and feed. The experimental diets were based on corn, wheat, extracted soybean meal, sunflower oil, limestone, and a premix with vitamins, minerals, amino acids, salt and sodium bicarbonate. The dietary treatments were 1) basal diet (control); 2) basal diet supplemented with natural betaine (1,000 mg of active n-betaine/ kg of feed) (ActiBeet, 97% betaine, Agrana Sales & Marketing GmbH, Vienna, Austria); 3) basal diet supplemented with synthetic betaine-HCL (1,000 mg of active syn-betaine-HCL (76 % betaine)/ kg of feed). Chicks were fed for 6 consecutive weeks with the starter feed from d 1 to 10, grower feed from day 11 to 24 and finisher feed from d 25 to 42. Representative feed samples for each group were analysed for determining the concentration of betaine in the diets. At least twice per day, all animals were controlled for adverse clinical signs to ensure their health and welfare. At 21, 28, and 35 d of age, five birds from each group were euthanized by injection of thiopental (20 mg/kg) into the wing vein and by bleeding of the jugular vein. Furthermore, the remaining birds were killed between 21 to 28, 28 to 35, and 35 to 42 days of age, respectively, for Ussing chamber measurements.

### Assessment of Birds’ Performance

Body weight (**BW**) was determined as an individual measurement at weekly intervals, on d 1, 7, 14, 21, 28, and 35 and the body weight gain (**BWG**) was calculated as the difference between the final and initial body weight during each of the intervals. Average daily gain (**ADG**) was calculated by dividing the weight gain by days. This was based on the equation:

$$\text{ADG}=\frac{\text{Finish}\;\text{weight}-\text{Start}\;\text{weight}}{\text{days}}$$

Furthermore, feed intake (**FI**) per pen (replicate) over the course of the experiment was determined as the difference between the amount of feed offered and the left over. Feed conversion ratio was calculated by dividing FI with BW gain. The average daily feed intake (**ADFI**) per treatment during the experiment was calculated by dividing the sum of feed intake (per pen) by days. The feed conversion ratio (**FCR**) FCR for each pen was estimated based on the ratio between the average feed intake per bird and weight gain.

### Histomorphological Investigation

For histomorphological examination, tissue samples were taken from jejunum close to the junction of Meckel's diverticulum and cecum. The samples were fixed in 4% formaldehyde. The processing consisted of serial dehydration, clearing, and impregnation with wax. Tissue cross-sections (5 per bird, 5-µm thick) from each of 5 birds per treatment were cut by a microtome and fixed on slides. Afterwards, deparaffination was performed in xylene (2 times 5 min) followed by rehydratation in alcohol 100% (5 min), 96% (5 min), and 70% (5 min). A routine staining procedure was carried out using hematoxylin and eosin. The sections were inspected under an Olympus BX53 microscope and documented with an Olympus DP72 camera (Olympus Corporation, Tokyo, Japan). For each sample, 5 replicates/bird, of intact well-oriented crypt-villus units were selected. Villus height was measured from the tip of the villus to the villus-crypt junction, whereas crypt depth was defined as the depth of the invagination between adjacent villi, and villus width was assessed at the middle of villi. Villus surface area was calculated from villus height and width at half height, and the villus height to crypt depth (**H:D**) ratio was also calculated as previously described (Awad et al., 2015a). The measurement was done with Cell Sens Image Program (Olympus Corporation).

### Jejunal and Cecal Barrier Function

The intestinal segments were immediately taken from the mid-jejunum and cecum after killing of birds (6 birds/group). Manipulation and experimental procedures were performed in accordance with Awad et al. (2019). Briefly, the intestinal segments were placed into ice-cold buffer solution (contained in mmol/L): NaCl, 115; KCl, 5; CaCl_2_, 1.5; MgCl_2_, 1.2; NaH_2_PO_4_, 0.6; Na_2_HPO_4_, 2.4; L-glutamine, 1; Na-D/L-lactate, 5; HEPES-free acid, 10; NaHCO_3_, 25; and mannitol, 10; pH 7.4) oxygenated with carbogen (95% O_2_/5% CO_2_) until they were mounted in the Ussing chamber. The intestinal segments were opened along the mesenteric border and washed free of intestinal content with cold buffer solution. The underlying serosal layer was stripped off and the epithelial sheets were mounted in Ussing chambers with an exposed area of 1.1 cm^2^. The Ussing chamber allows separate incubation of the mucosal and serosal side in an aerated buffer solution of the selected tissue. The serosal and mucosal surfaces of the tissues were bathed in12 mL of buffer solution. The bathing solutions were oxygenated (95% O_2_/ 5% CO_2_) and circulated in water-jacketed reservoirs maintained at 38°C. Flux rates of mannitol (J_man_) were measured at a bilateral concentration of 10 mM. The radioactive tracer, ^14^C-mannitol (0.1mCi/ml; Hartmann Analytic), was added to the mucosal solution. After a 40-min equilibration period, standards were taken from the mucosal side of each chamber and a 30-min flux period was established by taking 0.6-mL from the serosal compartment. Hot samples (100 μL) were collected at the beginning and end of the entire sampling period, whereas cold samples (600 μL) were collected at the start of each flux period. Epithelia were incubated for 3 h divided into the following three flux periods: 1) Baseline measurement period; 2) Second flux period, increasing the luminal ^14^C mannitol concentration from 10 mM to 20 mM and 3. Persistent period. After adding liquid scintillation fluid (Aquasafe 300 Plus, Zinsser Analytic, Maidenhead, UK) to the collected samples, the ^14^C mannitol presence was estimated by measuring the beta emission in a liquid scintillation counter. Unidirectional ^14^C mannitol fluxes from mucosa to serosa (J_ms_) were calculated from the net appearance of ^14^C in the serosal bathing solution overtime by using the mathematical procedure described previously (Ruhnau et al., 2020, 2021).

### Gene Expression Analysis (Real-Time qPCR) of Selected Tight Junction, Mucin, and Cytokines

For total RNA extraction approximately 25 mg from the stored intestinal samples (jejunum and cecum) were individually homogenized using Tissue Lyser (Qiagen, Hilden, Germany) (2x at 30 Hz for 2 min) and RNA extraction was performed according to manufacturer's instruction using the RNeasy Plus Mini Kit (Qiagen). After the initial extraction, RNA was cleaned using DNaseI (Qiagen) and RNase out (Thermo Fisher Scientific, Vienna, Austria) and stored at −80°C until further use. The concentration and purity of RNA were measured using A260/280 and A260/230 ratios by Nanodrop2000 spectrophotometry (Thermo Fisher Scientific, Vienna, Austria). RNA integrity (RIN) was analysed for all samples by Bioanalyzer_2100 (Agilent Technologies, Waldbronn, Germany) using RNA 6000 Nano Kit (Agilent Technologies).

Based on TaqMan probe chemistry, one-step RT-qPCR was performed using the Brilliant III Ultra-Fast QRT-PCR master mix kit (Agilent Technologies, Waldbronn, Germany). Amplification and quantification of the mRNA were performed using the AriaMx real-time PCR system (Agilent Technologies, Waldbronn, Germany) and the Agilent AriaMx1.5 software (Agilent Technologies). The thermal cycle profile for RT-qPCR was customized as follows: the reverse transcription phase for 10 min at 50°C followed by the hot start phase at 95°C for 3 min and 40 cycles of amplification at 95°C for 5 s and 60°C for 30 s.

Primer and probe concentrations were chosen according to previous standardisation protocols (von Buchholz et al., 2021). All samples were run in duplicate. Additionally, each sample was tested for a possible genomic DNA contamination and ran in duplex without reverse transcriptase for each gene of interest. Gene-specific sequences for primers for the genes of interest (CLDN-1, -5, OCLN, ZO-1, MUC-2 and cytokines: IL-1β, IL-10, Il-13, IFN-γ, TLR-4) were derived from sequence information in GeneBank as described (von Buchholz et al., 2021, 2022). Two replicates per sample were analyzed with normalization to suitable reference genes (TBP and RPL-13).

### Statistical Analysis

Data are presented as means with standard error of mean (**SEM**). The Kolmogorov-Smirnov test was used to test the normal distribution of the data before statistical analysis was performed. For performance, histology, mannitol flux, and mRNA gene expression, a multivariate general linear model followed by ANOVA, Duncan´s multiple range test and LSD were used for comparisons between groups. Differences were considered significant at a level of *P* ≤ 0.05. Statistical analyses were performed using IBM SPSS (version 24, SPSS Inc., Chicago, IL).

## RESULTS

### Effect of Natural Betaine and Synthetic Betaine-HCl on Growth Performance

All birds appeared clinically normal during the entire feeding trial. No mortality occurred over the course of the whole experiment. However, there were slight gross pathological lesions (congestions, necrotic foci, thickening of the intestinal wall) during necropsy of the birds supplemented with syn-betaine‐HCL.

**Figure 1:**
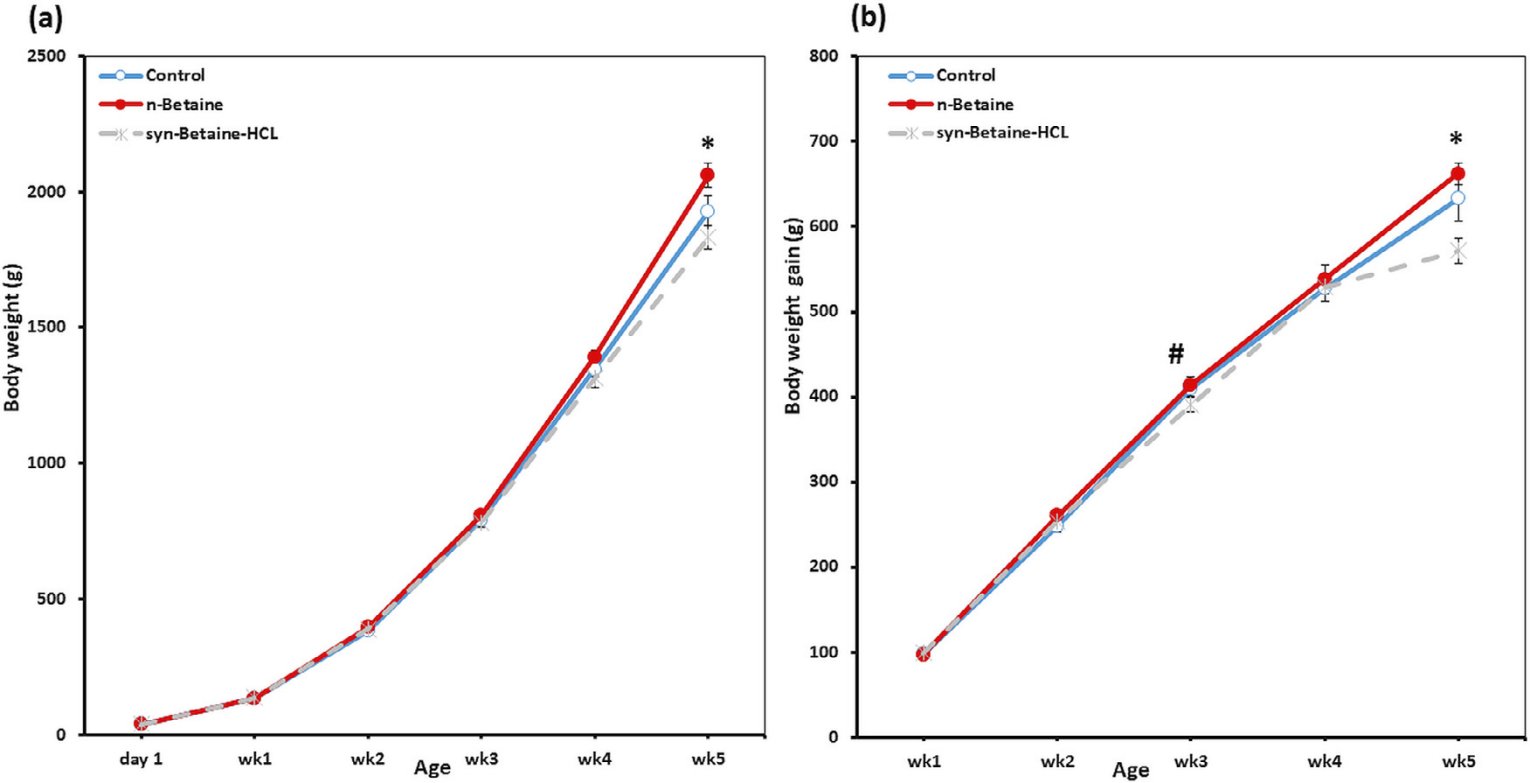
Effect of feed supplementations with betaine or betaine‐HCL on (a) body weight (BW) and (b) body weight gain (BWG). Results are presented as mean values and standard error of mean (SEM). Asterisks mark differences with *P* ≤ 0.1 (#), or *P* ≤ 0.05 (*).

**Figure 2:**
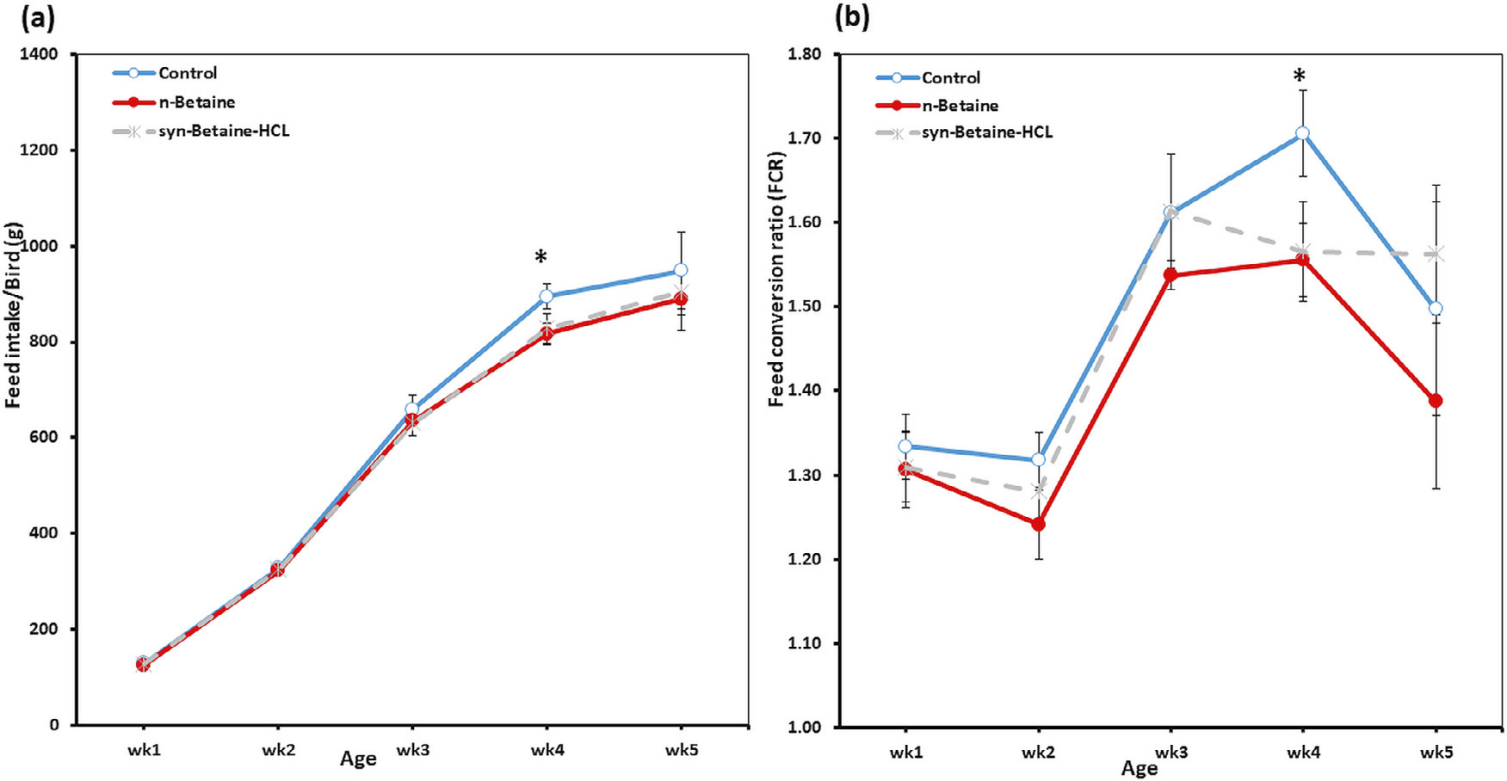
Effect of feed supplementations with betaine or betaine‐HCL on (a) feed intake (FI) and (b) feed conversion rate (FCR). Results are presented as mean values and SEM. Asterisks mark differences with *P* ≤ 0.05 (*).

The performance data during the growing period, from d 1 to d 35 of age, of the broiler chickens were evaluated weekly and results are shown in Figures 1 and 2. The initial body weight (BW) of chicks did not differ (*P* > 0.05) between the groups. At the end of the experiment week 5 (wk 5), the BW did not differ (*P* > 0.05) between the control birds and birds supplemented with syn-betaine‐HCL. However, birds supplemented with the n-betaine had a higher BW (*P* < 0.05) compared to the other groups. Additionally, the mean BW gain and the overall body weight gain were higher for broilers fed diet supplemented with n-betaine compared with the other groups. In contrast, a significant decrease in BWG was observed in the betaine-HCL group compared to the control and n-betaine groups (*P* = 0.056, *P* = 0.018, respectively).

The feed intake was not significantly different between groups during the first 3 wk (Figure 2A). However, a significant (*P* < 0.05) decreased feed intake was determined in birds fed with either natural or synthetic betaine compared to the control birds at wk 4. Throughout the whole trial, the average feed conversion ratio was lower in the group in which birds received n-betaine compared with the other groups, but this effect reached only the statistical significance at wk 4 (Figure 2B), indicating that the supplementation of n-betaine was able to improve the feed efficiency and the growth performance of broilers.

### Effect of Natural Betaine and Synthetic Betaine-HCl on Intestinal Histomorphology

**Figure 3:**
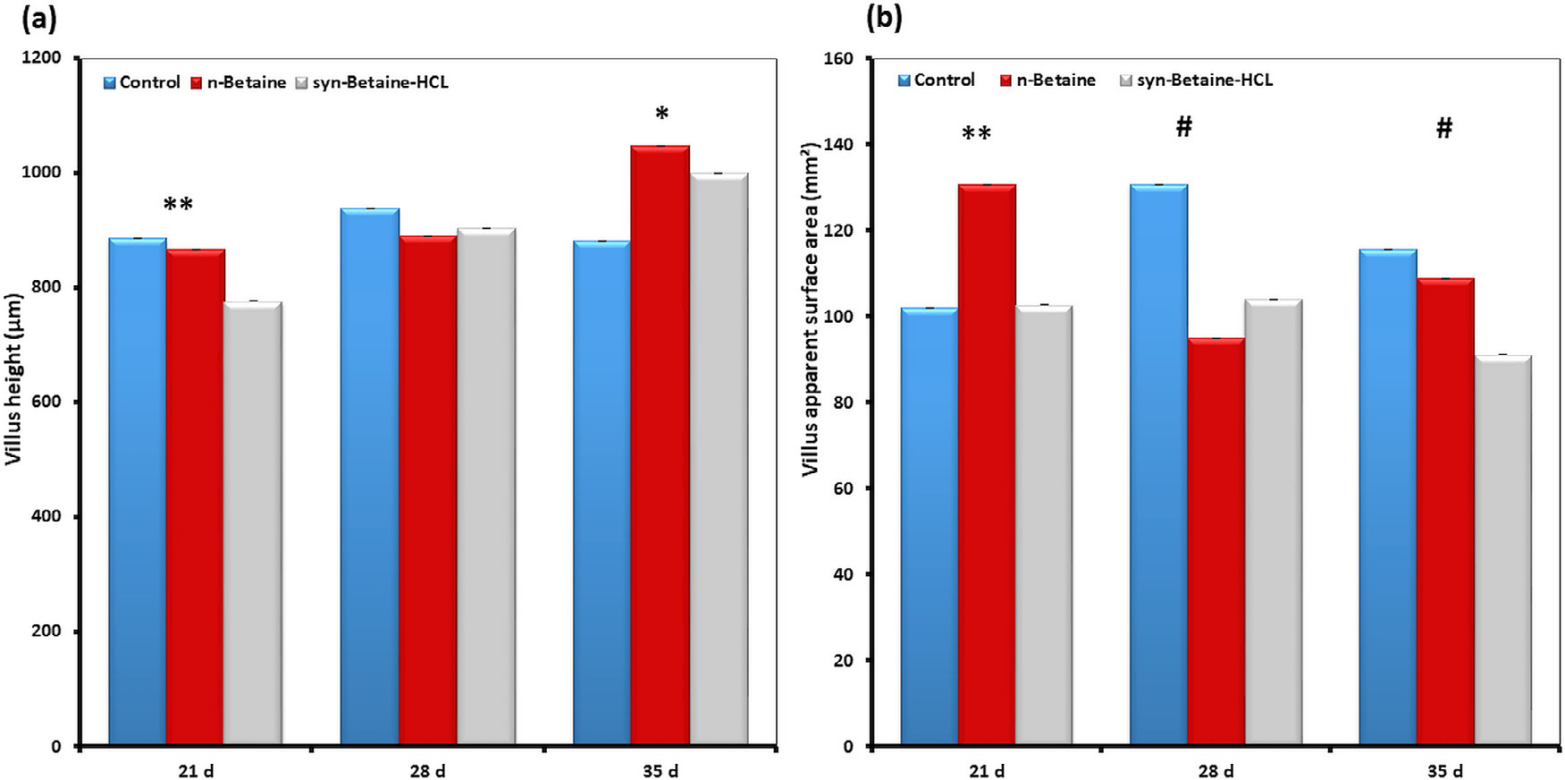
Effect of feed supplementations with betaine or betaine‐HCL on (a) Villus height (μm) and (b) Villus apparent surface area (mm^2^). Results are presented as mean values and SEM (n = 5). Asterisks mark differences with *P* ≤ 0.1 (#), *P* ≤ 0.05 (*), or *P* ≤ 0.01 (**).

**Figure 4:**
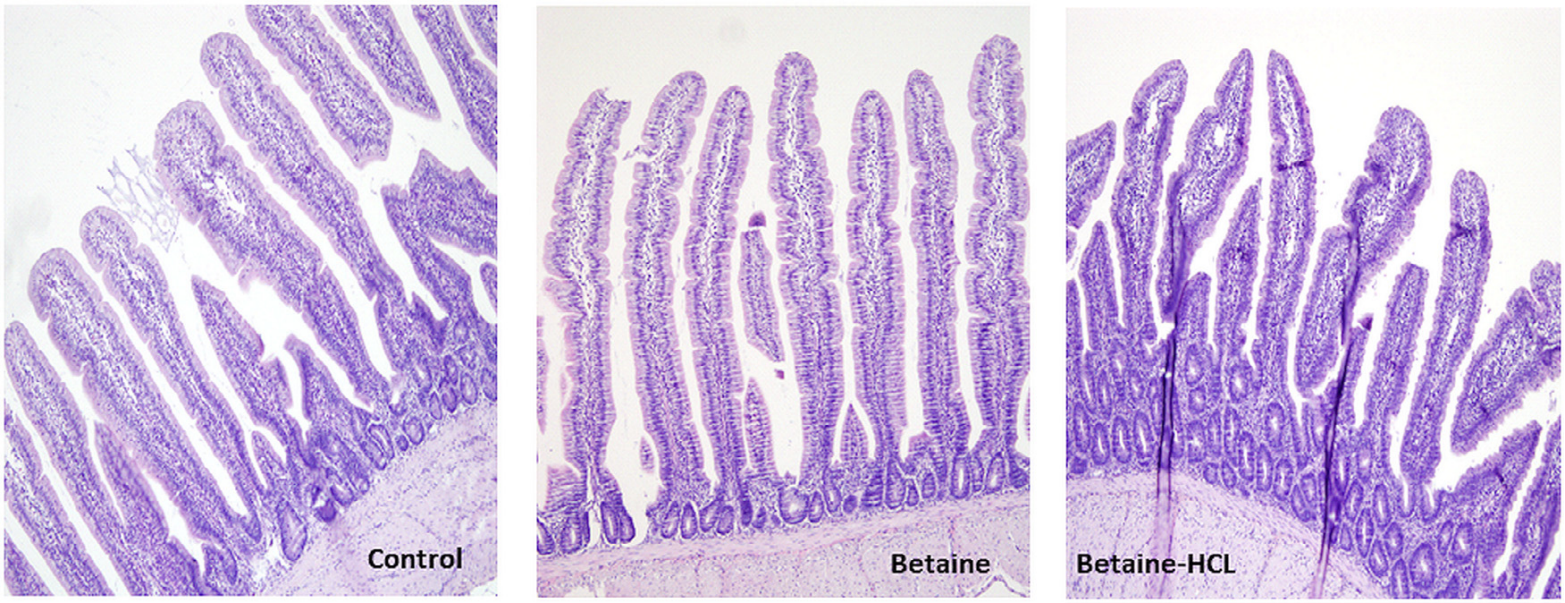
Histomorophology of jejunal villi of a 3-wk-old broiler chickens fed diets with either control, betaine or betaine-HCL. Sections were stained with hematoxylin and eosin. The image acquisition phase was done with a 10x magnification objective.

**Figure 5:**
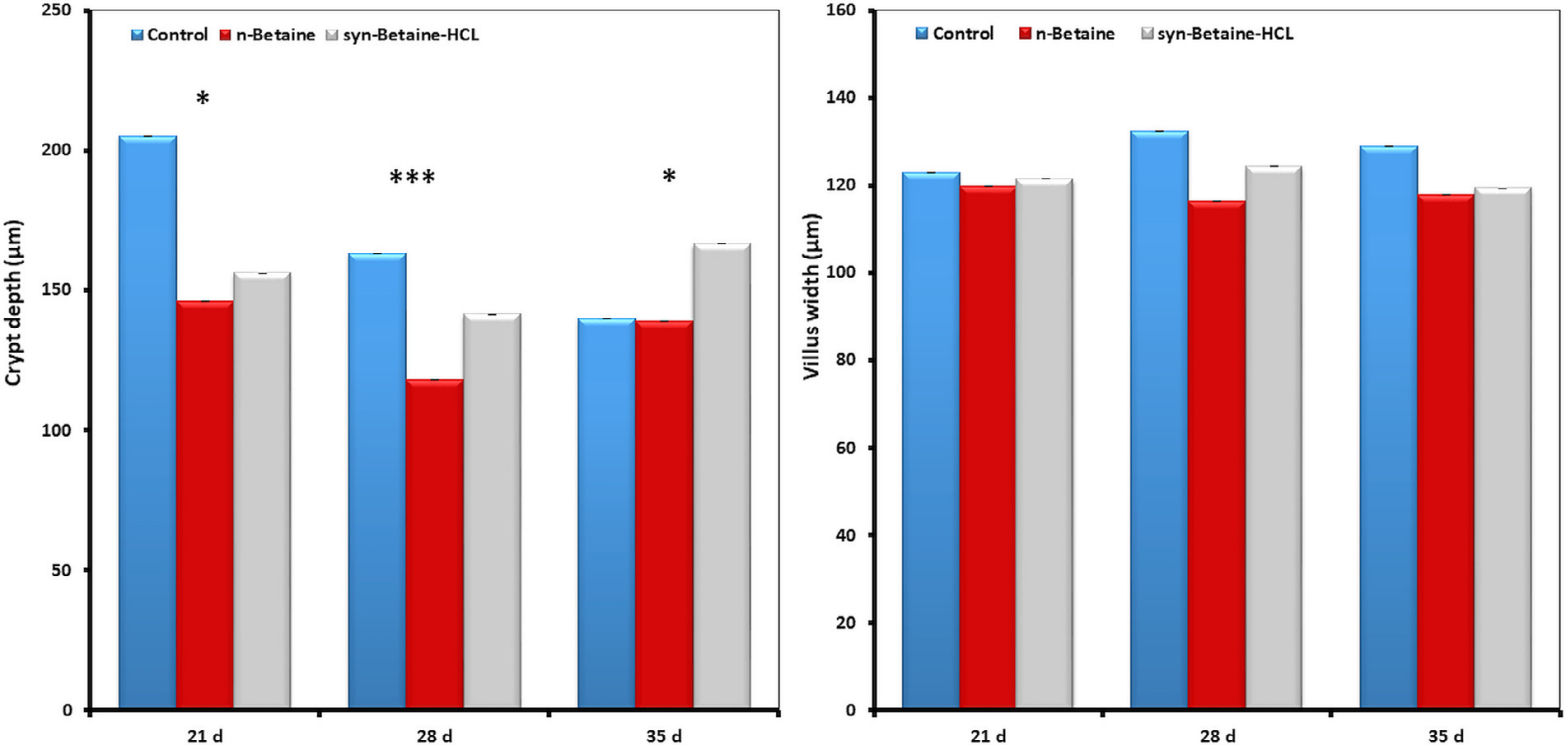
Effect of feed supplementations with betaine or betaine‐HCL on (a) Crypt depth (μm) and (b) Villus width (μm). Results are presented as mean values and SEM (n = 5). Asterisks mark differences with *P* ≤ 0.05 (*), or *P* ≤ 0.001 (***).

**Figure 6:**
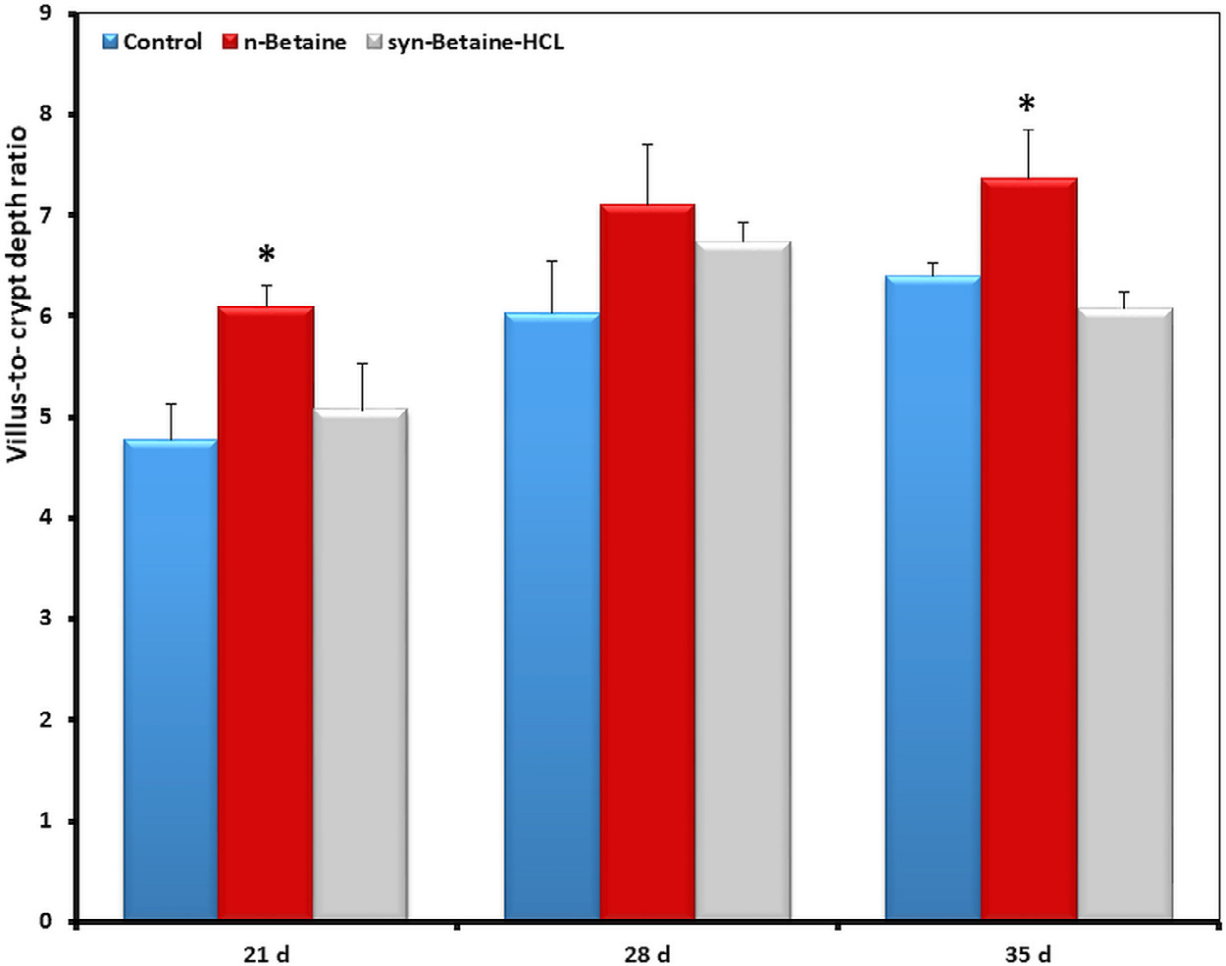
Effect of feed supplementations with betaine or betaine‐HCL on villus height: crypt depth ratio. Results are presented as mean values and SEM (n = 5). Asterisks mark differences with *P* ≤ 0.05 (*).

Histomorphometric analysis of the jejunum revealed that betaine supplementation altered the architecture of the small intestine (Figure 3, Figure 4, Figure 5, Figure 6). At 21 d of age, the villi in the jejunum were significantly (*P*  = 0.006) shorter (777 µm) in the syn-betaine-HCL group than in the controls (887 µm) and in the n-betaine group (867 µm, *P*  = 0.019; Figure 3). Syn-betaine-HCL supplementation also resulted in a tender decreased (*P*  = 0.070) villus surface area at 35 days of age (91 mm^2^) compared with controls (116 mm^2^). No effect of the syn-betaine-HCL was apparent for villus width. Furthermore, the jejunal crypt depth for both betaine treatment groups was significantly lower (*P* < 0.05) than the control at 21 and 28 d of age. In contrast, the crypt depth for the betaine consuming birds was significantly greater (*P* < 0.05) than the control at 35 d of ages (Figure 5).

The results of this study showed also that the villus height was higher in 35-day old chickens fed the supplemental n-betaine additive, yet this trend was not determined in 21- and 28-day-old chickens. However, the villus surface area was higher in 21-day-old chickens fed with the feed supplemented n-betaine, but this trend was not determined in 28- and 35-day-old chickens. Interestingly, at all-time points, there was a trend for a greater villus height to crypt depth ratio in the n-betaine group, indicating a rapid cellular migration to permit renewal of the villus (Figure 6).

### Effect of Natural Betaine and Synthetic Betaine-HCl on Intestinal permeability

**Figure 7:**
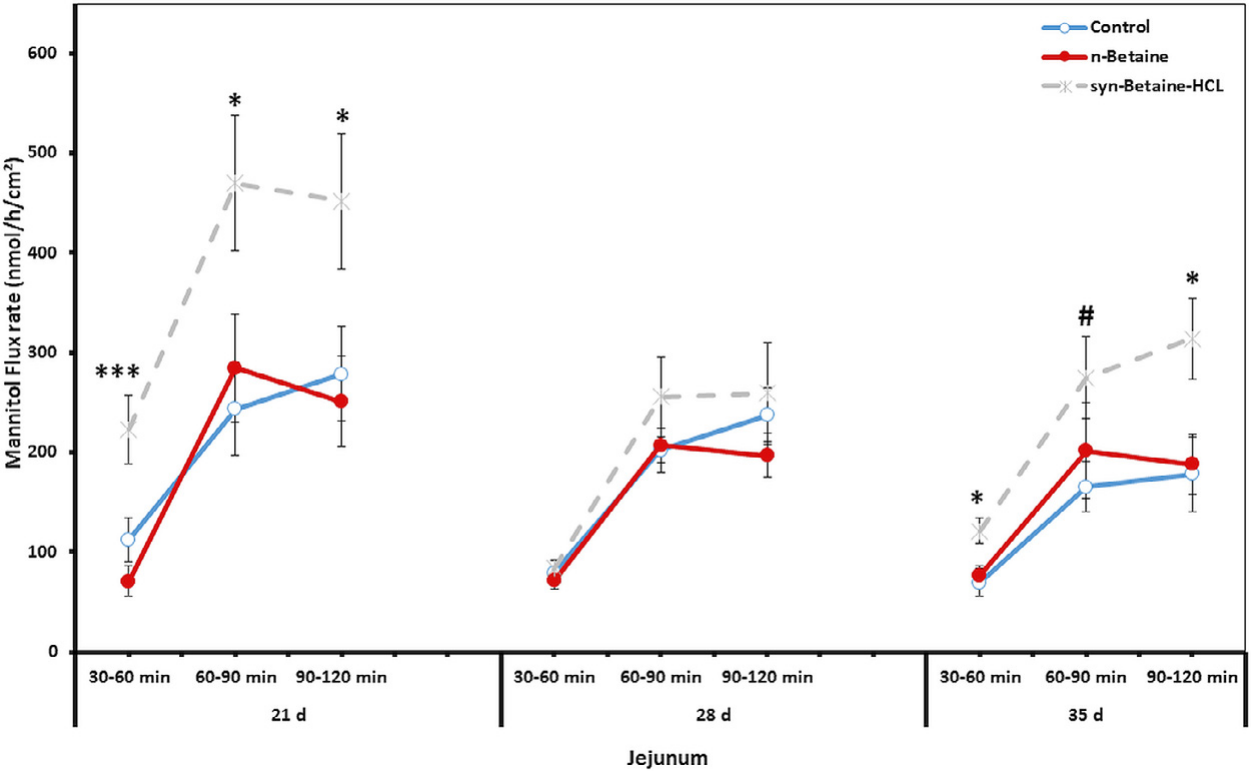
Effect of betaine and betaine‐HCL on paracellular permeability in jejunum at different time points. Mucosal to serosal flux (Jms) of the permeability marker 14C‐mannitol were performed in Ussing chambers. Data are presented as the mean values and SEM (n = 6). Asterisks mark differences with *P* ≤ 0.1 (#), *P* ≤ 0.05 (*), or *P* ≤ 0.001 (***).

**Figure 8:**
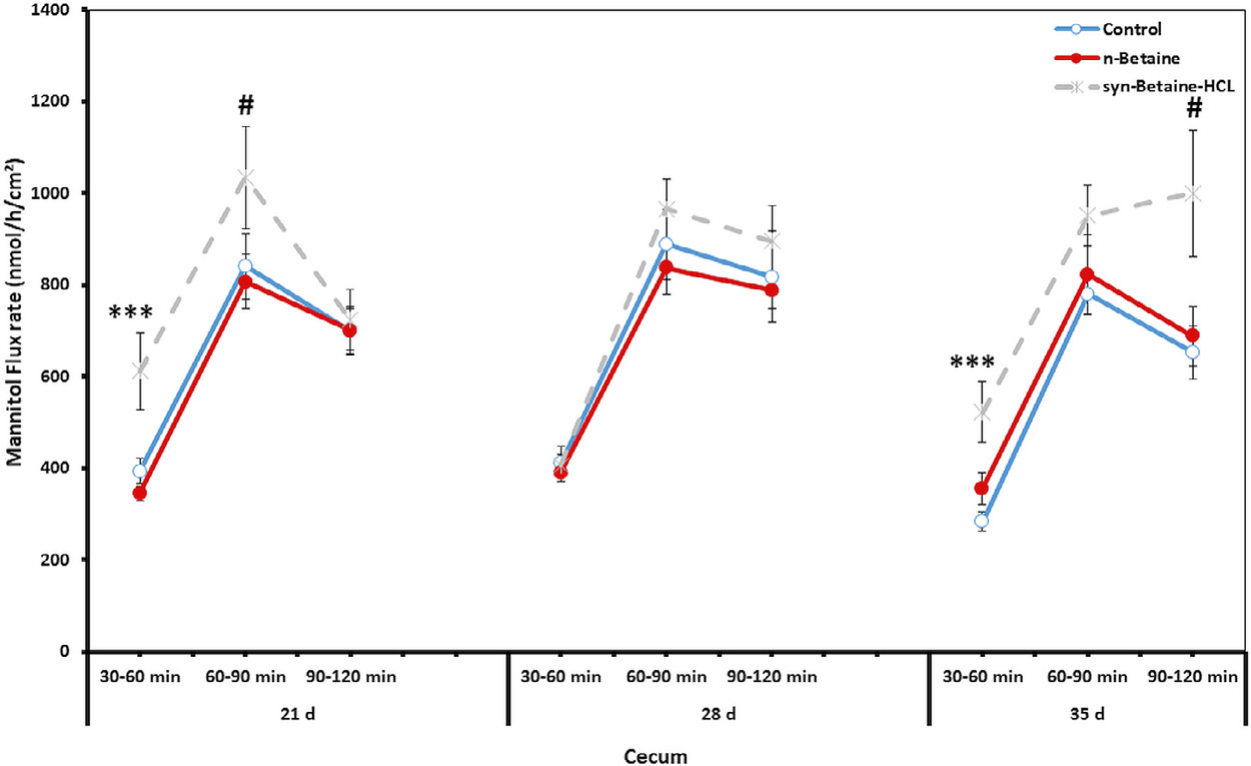
Effect of betaine and betaine‐HCL on paracellular permeability in cecum at different time points. Mucosal to serosal flux (Jms) of the permeability marker 14C‐mannitol were performed in Ussing chambers. Data are presented as the mean values and SEM (n = 6). Asterisks mark differences with *P* ≤ 0.1 (#), or *P* ≤ 0.001 (***).

Fluxes of the paracellular marker molecule, ^14^C mannitol, were used in order to elucidate whether permeation via the paracellular epithelial pathway was altered by betaine. The unidirectional flux of ^14^C mannitol in jejunum and cecum is shown in Figures 7 and 8. The results revealed that syn-betaine-HCL supplementation induced a significant increase in the ^14^C mannitol flux in the jejunum and cecum in all flux periods, indicating an increased paracellular leakage. During the baseline period, there were significant differences (*P* < 0.001) in the flux of the marker molecule in both jejunum and cecum among the groups at 21 d of age. Furthermore, during the second and the third flux periods (from 60 to 90 min), a continuous increase in ^14^C mannitol flux was found. Nevertheless, the impact of the syn-betaine-HCL supplementation was much more persistent, in either jejunum or cecum at 21 and 35 d of age compared with 28 d of age, as there were significant differences throughout the flux periods.

On the contrary, feeding of n-betaine induced no significant changes in intestinal permeability and led to a comparable permeability of the chicken gut as in the control group. The difference in the mannitol flux between the n-betaine and syn-betaine-HCL fed group was more pronounced in the jejunum and cecum at 21 and 35 d of age, but this difference did not reach statistical significance at 28 d of age. Furthermore, the results showed that the impact of dietary treatments on intestinal permeability varies highly between different gut sites, as a significant difference was noticed in mannitol fluxes in the cecum compared to the jejunum, which likely reflects different mechanisms for changes in intestinal permeability of each intestinal segment.

### Effect of Natural Betaine and Synthetic Betaine-HCl on Intestinal Gene Expression

**Figure 9:**
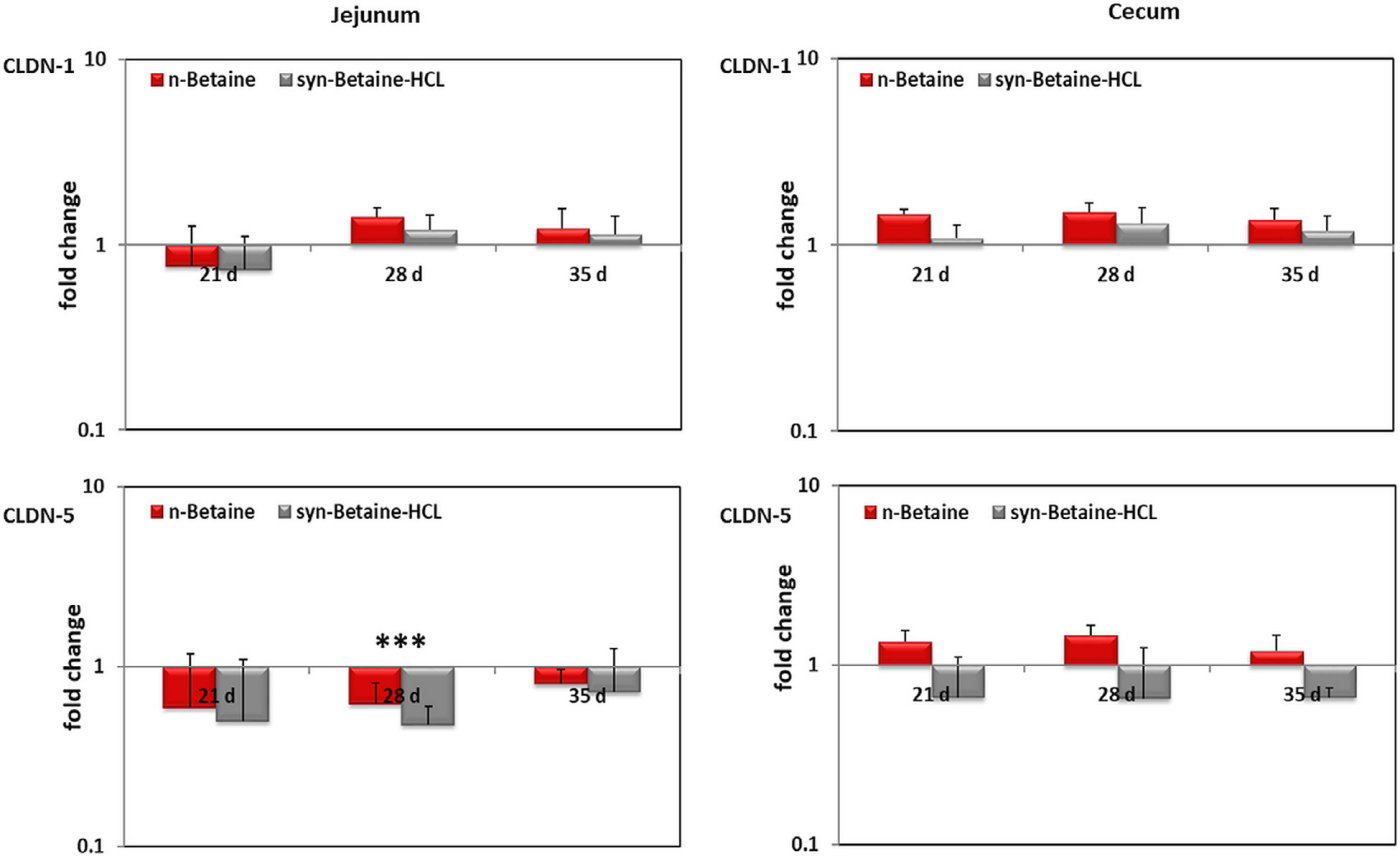
CLDN-1 and CLDN-5 mRNA expression levels measured by reverse transcription‐qPCR. The expression levels in jejunum and cecum are shown as fold change of mRNA expression levels relative to age‐matched controls (n = 5). Asterisks mark differences *P* < 0.001 (***).

**Figure 10:**
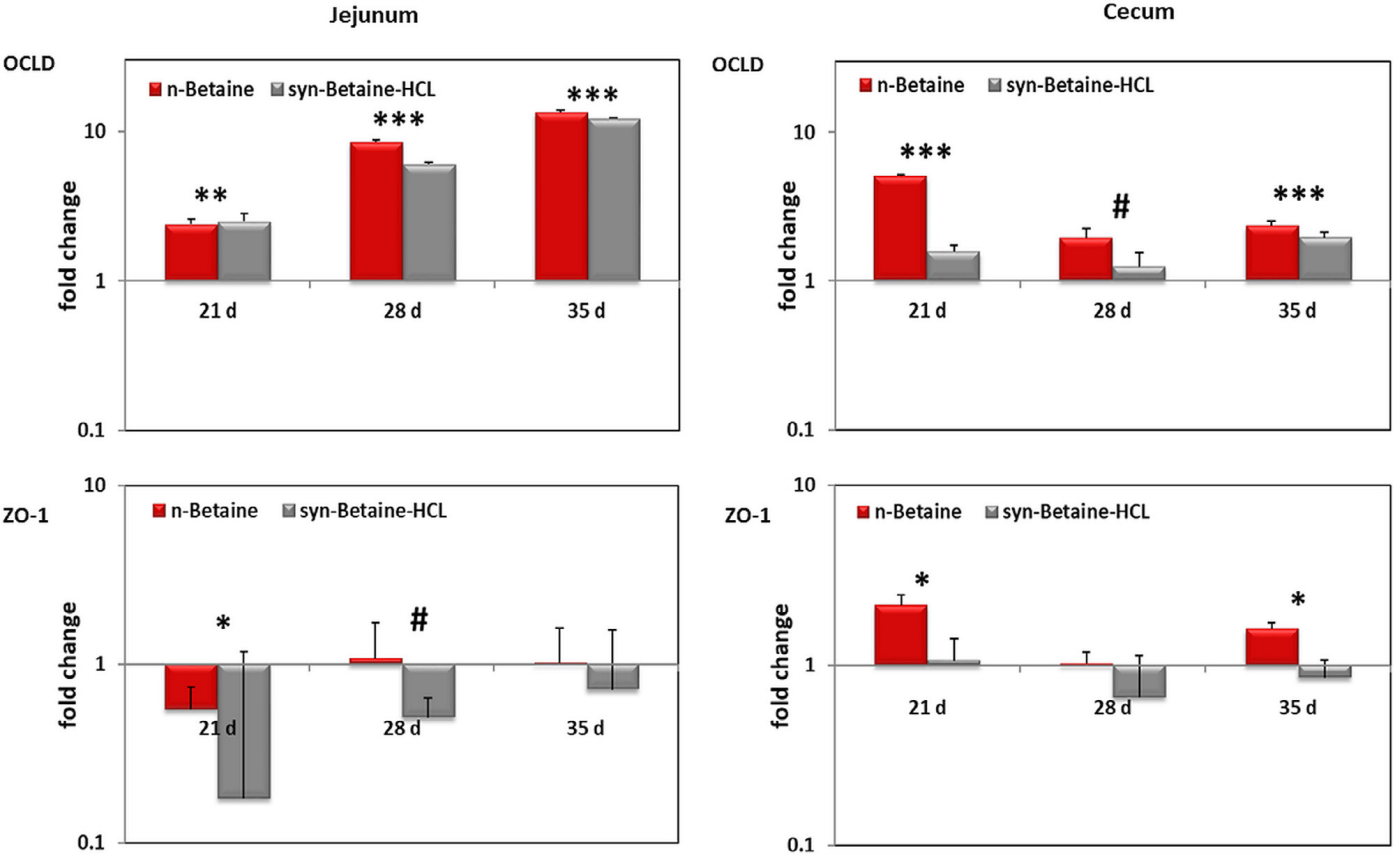
OCLN and ZO-1 mRNA expression levels measured by reverse transcription‐qPCR. The expression levels in jejunum and cecum are shown as fold change of mRNA expression levels relative to age‐matched controls (n = 5). Asterisks mark differences *P* < 0.1 (#), *P* < 0.05 (*), *P* ≤ 0.01 (**), or *P* < 0.001 (***).

To investigate whether the increased intestinal permeability observed in syn-betaine-HCL fed birds was associated with altered TJ gene expression, TJ mRNA expression was examined by qRT-PCR. For normalization, RPL-13 (ribosomal protein L-13) and TBP (TATA box binding protein) were used as reference genes since they gave very low expression variation in both jejunum and cecum. As shown in Figures 9 and 10, syn-betaine-HCL significantly (*P* < 0.001) decreased the gene expression of the barrier-forming TJ protein (CLDN-5) in jejunum at 28 days of age with a tendency to decrease in cecum, concomitant with no change of the barrier-tightening CLDN-1 in both jejunum and cecum. In contrast, in the jejunum, feeding of either n-betaine or syn-betaine-HCL increased the expression of OCLN at all-time points. Similarly, in the cecum, the expression of OCLN was significantly upregulated in n-betaine fed birds at all-time points but only upregulated in syn-betaine-HCL fed birds at 35 d of age. Likewise, feeding of syn-betaine-HCL (*P* ≤ 0.05) decreased the expression of ZO-1 early in the jejunum at 21 d of age and later in cecum at 35 days of age.

**Figure 11:**
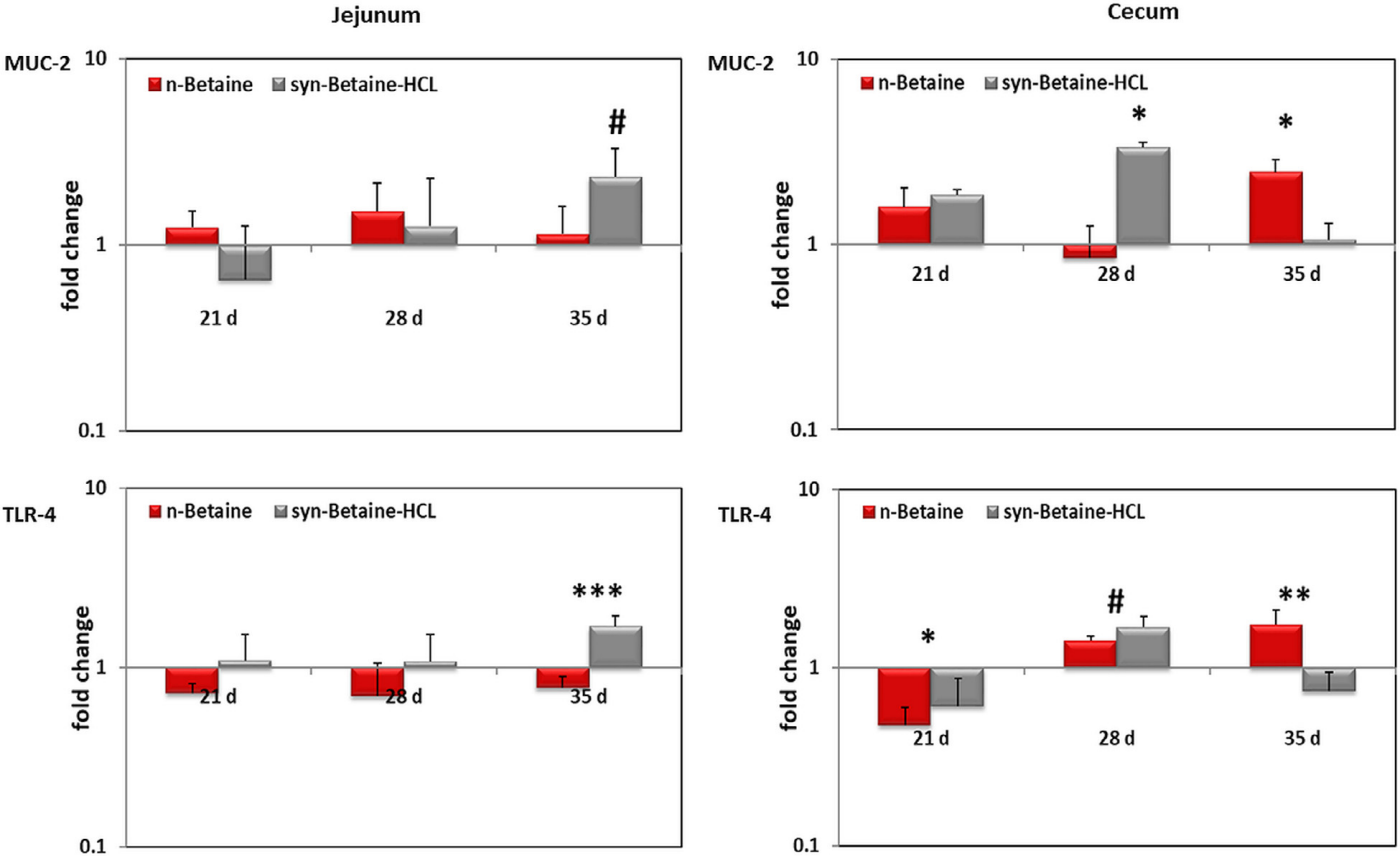
MUC-2 and TLR‐4 mRNA expression levels measured by reverse transcription‐qPCR. The expression levels in jejunum and cecum are shown as fold change of mRNA expression levels relative to age‐matched controls (n = 5). Asterisks mark differences *P* < 0.1 (#), *P* < 0.05 (*), *P* ≤ 0.01 (**), or *P* < 0.001 (***).

In jejunum, expression of MUC-2 was similar between the controls and treated birds. In cecum, however, the relative expression of the MUC-2 gene showed a significant upregulation (*P* ≤ 0.05) in the n-betaine group compared to the syn-betaine-HCL group at the age of 35 d (Figure 11). An opposite result was obtained at 28 d of age, when the expression of MUC-2 was upregulated in the syn-betaine-HCL group compared to the n-betaine group. The mRNA expression data were consistent with the intestinal permeability results as there was no difference between groups at the age of 28 d.

Furthermore, the potential immunomodulatory effects of betaine on innate and antibody-mediated immune response in chickens were investigated. Interleukin (**IL**)-1β gene expression as a pro-inflammatory cytokine, IL-13 as a Th2 cytokine, interferon (**IFN**)-γ as a Th1 cytokine and Toll-like receptor (**TLR**)-4 as an inducer of innate immune responses were measured. A significant upregulation in expression of TLR-4 on day 35 of age in birds supplemented with n-betaine was noticed (Figure 11).

**Figure 12:**
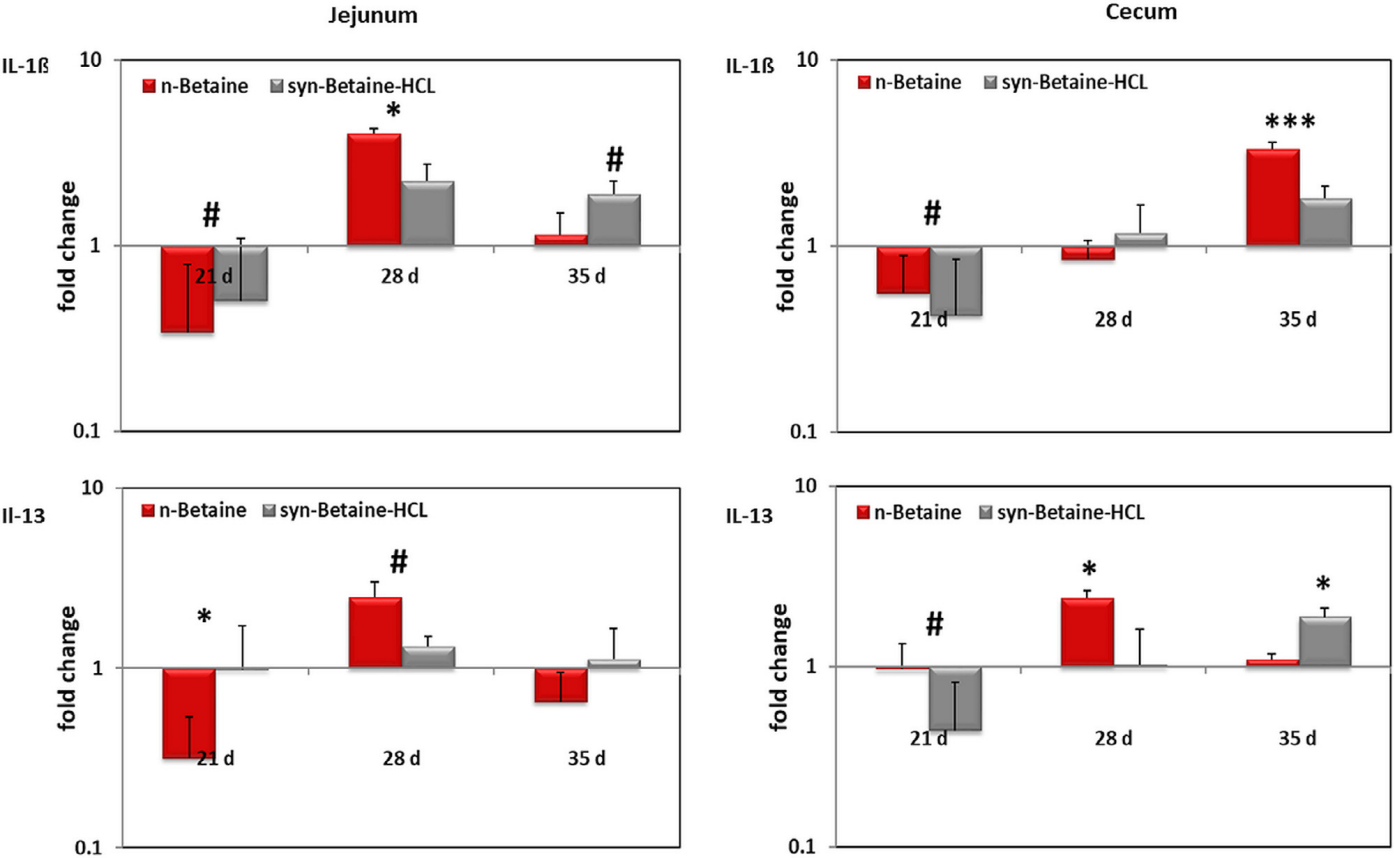
IL‐1ß and IL‐13 mRNA expression levels measured by reverse transcription‐qPCR. The expression levels in jejunum and cecum are shown as fold change of mRNA expression levels relative to age‐matched controls (n = 5). Asterisks mark differences *P* < 0.1 (#), *P* < 0.05 (*), or *P* < 0.001 (***).

**Figure 13:**
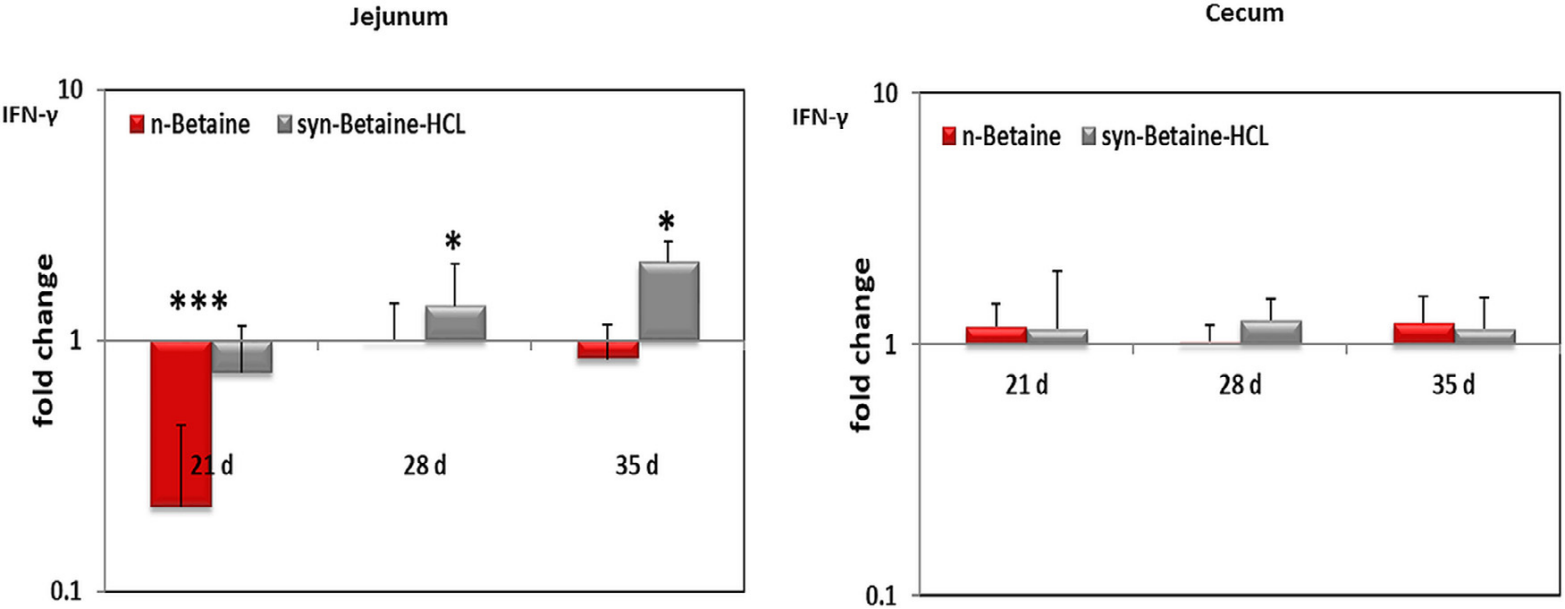
INF‐γ mRNA expression levels measured by reverse transcription qPCR. The expression levels in jejunum and cecum are shown as fold change of mRNA expression levels relative to age‐matched controls (n = 5). Asterisks mark differences *P* < 0.05 (*), or *P* < 0.001(***).

Figure 12 shows that the n-betaine and syn-betaine-HCL supplemented birds’ markedly reduced Il-1β mRNA expression in both jejunum and cecum at 21 d of age. However, at the age of 28 d, both n-betaine and syn-betaine-HCL significantly increased Il-1β gene expression in jejunum. Furthermore, the expression of IL-13 in jejunum was significantly downregulated at 21 d of age and upregulated at 28 d of age in both jejunum and cecum of birds supplemented with n-betaine. However, in cecum, the expression of IL-13 was upregulated in syn-betaine-HCL group at the age of 35 d. Similarly, the expression of IFN-γ in jejunum was significantly downregulated at 21 d of age in birds supplemented with n-betaine and upregulated on d 28 and 35 of age in birds supplemented with syn-betaine-HCL (Figure 13).

## DISCUSSION

Improving broiler performance is still an essential task in animal production, especially under certain environmental challenges. The use of dietary supplements has been long attempted to achieve this goal. Betaine, a methyl group donor and osmolyte, plays a role in lipid metabolism by stimulating oxidative catabolism of fatty acids through carnitine synthesis (Craig, 2004). Betaine is a highly appreciated feed additive in the diet of many farm animals. Several studies have shown that betaine improved growth, feed conversion efficiency and breast yield (Wang et al., 2004; Attia et al., 2005; Waldroup et al., 2006; Hassan et al. 2011; Rao et al., 2011; dos Santosa et al., 2019). Nevertheless, in most of these studies, the betaine source is not declared. Betaine can be supplemented as natural betaine, isolated from natural sources or synthetic sources, as betaine-HCl. Little is known about the comparison between n-betaine and syn-betaine-HCl. Besides, there is no specific information about the underlying mechanisms and the optimal dietary inclusion level in order to optimize birds’ health, nutrient digestibility and to improve growth parameters. Within this study, we provide novel information about such effects of n-betaine and syn-betaine-HCl on jejunal and cecal integrity in chickens, as evidenced by alterations in intestinal morphology and permeability.

The results of the present study showed that the body weight did not differ between the control birds and birds supplemented with syn-betaine‐HCL. On the contrary, n-betaine supplementation has positive effects on performance, evidenced by improvement in body weight gain and feed conversion ratio. In line with this, Amerah (2014) found that natural betaine improved bird performance and nutrient digestibility better than synthetic betaine. A reasonable explanation for the increase in performance in n-betaine fed birds may be due to improved nutrient utilization, which increases the availability of sulfur-containing amino acids (methionine and cysteine) for protein deposition in muscle, and reduced requirement for metabolizable energy (Eklund et al., 2005). Furthermore, the adverse effect on BW in the syn-betaine-HCl group compared with n-betaine may be related to changes in gut physiology, gut microbiota, amino acid digestibility and changes of the intestinal pH caused by HCL, evidenced by suppressed villus length and a subsequent decrease in the nutrient absorption surface area in the jejunum (Awad et al., 2011). This can also be explained by the fact that the used concentration of active syn-betaine-HCl is higher (1,000 mg/ kg diet) compared to other studies that reported a positive effect of syn-betaine-HCL on bird performance. In addition, as a chemically synthesised product, betaine-HCl contains a high level of trimethylamine (**TMA**) as a by-product; however, this TMA is undetectable in a recent study using a natural betaine product (Amerah, 2014).

The epithelial surface area is one important criterion for the efficiency of nutrient absorption (Pluske et al., 1996). In this study, syn-betaine-HCl induced a decrease in the jejunal villus height and crypt depth, as well as a reduction of the villus absorptive area at 21 d. These changes point either to a decreased life span of enterocytes or to a compromised enterocyte renewal. However, the supplementation of natural betaine in the diet resulted in a significant increase in the length of the jejunal villi of chickens on d 35. Furthermore, the results showed that there were significant differences in the depths of jejunal villi crypts between the chickens from the experimental groups compared to the chickens from the control group. Within the explanation of betaine influence on the histomorphological features of chickens’ intestines, it is important to keep in mind that diet composition is in fact the main factor that can modify the histomorphology of the intestine, and consequently its absorptive capacity, which ultimately defines the growth performance of chickens (Awad et al., 2009). It has been shown that longer villi are associated with active cell mitosis, which provides a greater absorptive potential of villi for various nutrients and deeper intestinal crypts indicate a higher proliferative activity of tissue in order to allow the renewal of the intestinal villi (Onderci et al. 2006; Hamedi et al., 2011).

This study further showed that there was a statistically significant difference in the villus height to crypt depth ratio at different time points between the groups, suggesting that a higher ratio of villous height and crypt depth refers to a greater capacity of nutrient digestibility and absorption in chickens (Silva et al., 2009). Hamedi et al. (2011) reported that the shorter intestinal villi relative to crypt depth might point to a smaller number of absorptive cells and a larger number of goblet secretory cells. Secretory cells are responsible for the secretion of mucins that form a mucinous lining of the intestinal epithelium, thus increasing the number of secretory cells and leading to an increased secretion of mucin, which is supported by the present study. We also found that the expression of MUC-2 was upregulated in the syn-betaine-HCL group. Thus, changes in the quantity or composition of mucin of the intestinal mucosal surface can reduce the absorption of nutrients and/or increase the amount of energy required to maintain function of the intestines.

Apart from the morphological changes in the intestine of syn-betaine-HCL fed birds, the results showed that the impact of syn-betaine-HCL on intestinal permeability varied highly between different gut sites or certain time points, which likely reflects different mechanisms for the alterations of intestinal permeability of each intestinal segment. Interestingly, the intestinal permeability was not adversely affected by the feeding of n-betaine and resulted in a comparable intestinal permeability as in the control group. Effects of n-betaine and syn-betaine-HCl on gut permeability in chickens have never been studied.

Interestingly, in the current study, the increased paracellular permeability in birds supplemented with syn-betaine‐HCL was associated with a downregulation of the CLD-5 and ZO-1 mRNA expression in the small intestine and in the cecum, indicating an alteration of certain tight junction proteins most likely via distinct signal transduction pathways. The results indicate that the alteration of ZO-1 probably prevents its interaction with claudins and occludin which, consequently, may contribute to the disturbance of the TJ barrier and leads to enhanced intestinal permeability (Hamada et al., 2010). Furthermore, the increase in paracellular intestinal permeability induced by syn-betaine-HCl indicates that syn-betaine-HCl contains components that increase the paracellular permeability, which can promote the escape of harmful luminal substances to the basolateral side and thus trigger immune responses (Suzuki, 2013, Awad et al., 2015b). Similarly, Putaala et al. (2018) found that syn-betaine-HCl induced rapid declines in tight junction integrity and cellular ATP content after 1 h and 6 h and upregulated the inflammatory cytokines IL-6 and IL-8 after 24 h in human Caco-2 cells. Yegani and Korver (2008) reported that increasing paracellular permeability and luminal antigen flow in the body could trigger inflammatory reactions that could reduce the energy available for broiler growth and thus limit performance. The increase in intestinal permeability by syn-betaine-HCl can affect intestinal inflammation and thus be the reason for the reduced performance of the birds, which is shown in the current study. Moreover, we revealed that both n-betaine and syn-betaine-HCL had a similar effect on the mRNA expression patterns of OCLN, suggesting that occludin plays an important role in maintaining the integrity of the intestinal epithelial barrier and its’ upregulated expression in birds fed syn-betaine-HCL may therefore compensate for the loss barrier function. In this context it has been reported that the overexpression of occludin mRNA encoding TJ could be a result of a compensatory mechanism protecting the gastrointestinal tract from pathogenic bacteria and restoring intestinal permeability (Barekatain et al., 2019).

In the actual study, we also found that syn-betaine-HCl significantly upregulated the IFN-γ mRNA expression in the jejunum of chicken which may promote TJ permeability, as it was shown that IFN-γ plays an important role in intestinal barrier dysfunction (Yang et al., 2014). The present results also demonstrate that both n-betaine and syn-betaine-HCL upregulated the intestinal Il-1β mRNA expression. An explanation for this finding might be due to direct or indirect effects of betaine on changes in nutrient flow and gut microbiota, which play a crucial role in modulation of the gut epithelial barrier. Furthermore, the well-known function of the IL-1β as a pro-inflammatory cytokine activates and enhances the production of other cytokines and chemokines, such as interleukins -2 and -10, most likely antagonizing the action of pro-inflammatory cytokines on TJ permeability (Capaldo and Nusrat, 2009; Chen et al., 2016).

Syn-betaine‐HCL supplementation also resulted in downregulation of TLR‐4 in cecum at d 35 of age, suggesting that the greater intestinal permeability may be a TLR‐4‐dependent process. It was reported that TLRs play an important role in controlling crypt dynamics, enhancing epithelial barrier integrity and promoting immune tolerance towards the gut microbiota (Burgueño and Abreu, 2020). Our findings revealed that n-betaine had the potential to modulate the expression of certain TLRs and IL-1β and consequently enhance the immune response in chickens. Therefore, future studies should focus on elucidating the role of betaine under different challenges and health conditions.

Overall, based on differences in gut activity and function it can be concluded that natural betaine, vs. betaine hydrochloride (chemical synthesis), has a different potential to improve the productive performance and health status of broiler chickens.
